# Successfully Replanted An Amputated Hand: A Case Report

**DOI:** 10.7759/cureus.23366

**Published:** 2022-03-21

**Authors:** Khalid Murrad, Mohammed Ehsan Rashidi, Abdulla Altamimi

**Affiliations:** 1 College of Medicine, King Saud University, King Saud University Medical City, Riyadh, SAU; 2 Department of Plastic and Reconstructive Surgery, King Saud Medical City, Riyadh, SAU

**Keywords:** amputation, hand, hand amputation, microvascular, replantation

## Abstract

Hand amputation is known to be one of the most debilitating injuries. Its impact on the patient results in multiple diverse outcomes and affects daily life activities and career. With that in mind, we understand how devastating upper limb amputations can be. In this case, we report a middle-aged male who suffered an amputation at the level of the distal forearm and underwent replantation at King Saud Medical City in Riyadh, Saudi Arabia. Dealing with an amputated limb requires knowledge and awareness, starting with the patient and moving on to all healthcare providers involved.

## Introduction

Amputare is a Latin verb meaning to cut off or cut away [1]. Amputation is defined as removing a limb partially or totally by trauma or surgery [2]. Disarticulation is defined as the removal of a limb through a joint. The American Society for Surgery of the Hand has defined replantation or reattachment as “the surgical reattachment of a body part, most commonly a finger, hand or arm, that has been completely cut from a person’s body.” In 1962, a team led by Ronald Malt at Massachusetts General Hospital in Boston, Massachusetts, United States, performed the first-ever replantation of the brachial artery on a 12-year-old child [3]. In 1963, a team led by Zhong Wei Chen of the Sixth People’s Hospital in Shanghai reported a replantation using magnification to a machinist’s hand [4]. In 1963, Kleinert performed the first revascularization of a partially amputated finger [5]. In 1968, Komatsu and Tamai from Japan performed the first replantation of a digit [6]. It is essential to preserve the viability of the amputated part, which should be placed inside a sealed container situated within ice after it has been wrapped in a thin fabric of silk, linen, or cotton to avoid direct contact with ice, which might lead to permanent tissue damage [7]. Replantation has several determining aspects, such as the mechanism and level of injury, the significance of the amputated part, indications, and contraindications to attempt the replantation procedure.

The primary indications for reparations involve injuries that are through the palm, at wrist level or proximal to the wrist, and almost all parts in children. Relative indications include ring avulsion and injury through or above the elbow. Contraindications include previous severe trauma, a compromised function of the amputated part, severe crushing mechanisms, associated medical illnesses and other life-threatening injuries such as vascular disorders, prolonged ischemia time with muscle bulk (>6 hours), and segmental amputation. Any serious damage to tissue by severe trauma, which may lead to decreased vascularization, decreased healing mechanism, scarring, and increased rates of infection and contamination, results in poor outcomes in salvaging the part. Patients with associated medical illness are at risk of increased morbidity and mortality; these illnesses may include severe cardiac and/or pulmonary disease and anesthesia difficulties, which may contribute to the instability of the patient’s health, and must be addressed before considering replantation. Relative contraindications to replantation include prolonged warm ischemia time, medically unfit patient, disabling psychiatric illness, tissue contamination, and prolonged ischemia time with no muscle bulk (>12 hours). Warm ischemia by the term is the ischemia of cells and tissues under normothermic conditions. Prolonged warm ischemia time is more than six hours for sites where large muscles are present. Extending ischemia time can be achieved by cooling and prolonging ischemia time to 12 hours in significant limb replants [8].

We present an interesting case with a minor diversion of published literature as repairing one artery and two veins; in our patient, two arteries and one vein were repaired. We are proud to take you on a brief journey of our patient from his ED presentation and preparation and precautions that were taken preoperatively and intraoperatively and describe the details and surgical technique used, as well as, lastly, the postoperative care and management, including clinic follow-ups.

## Case presentation

**Figure 1:**
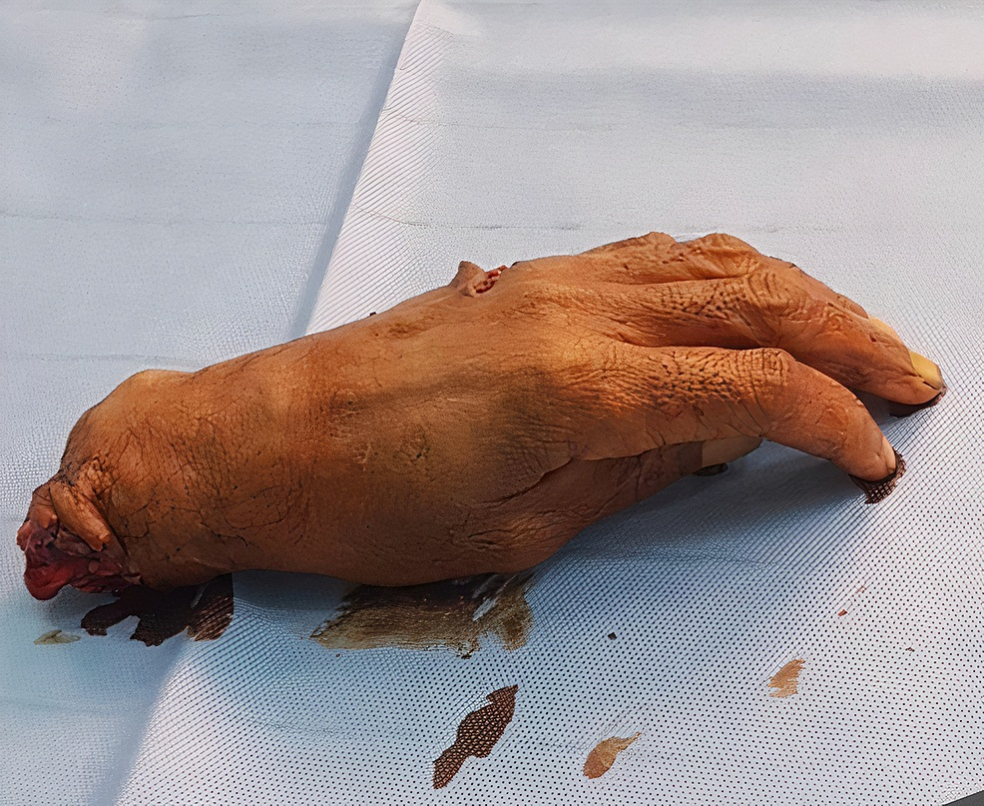
Amputated hand on presentation

**Figure 2:**
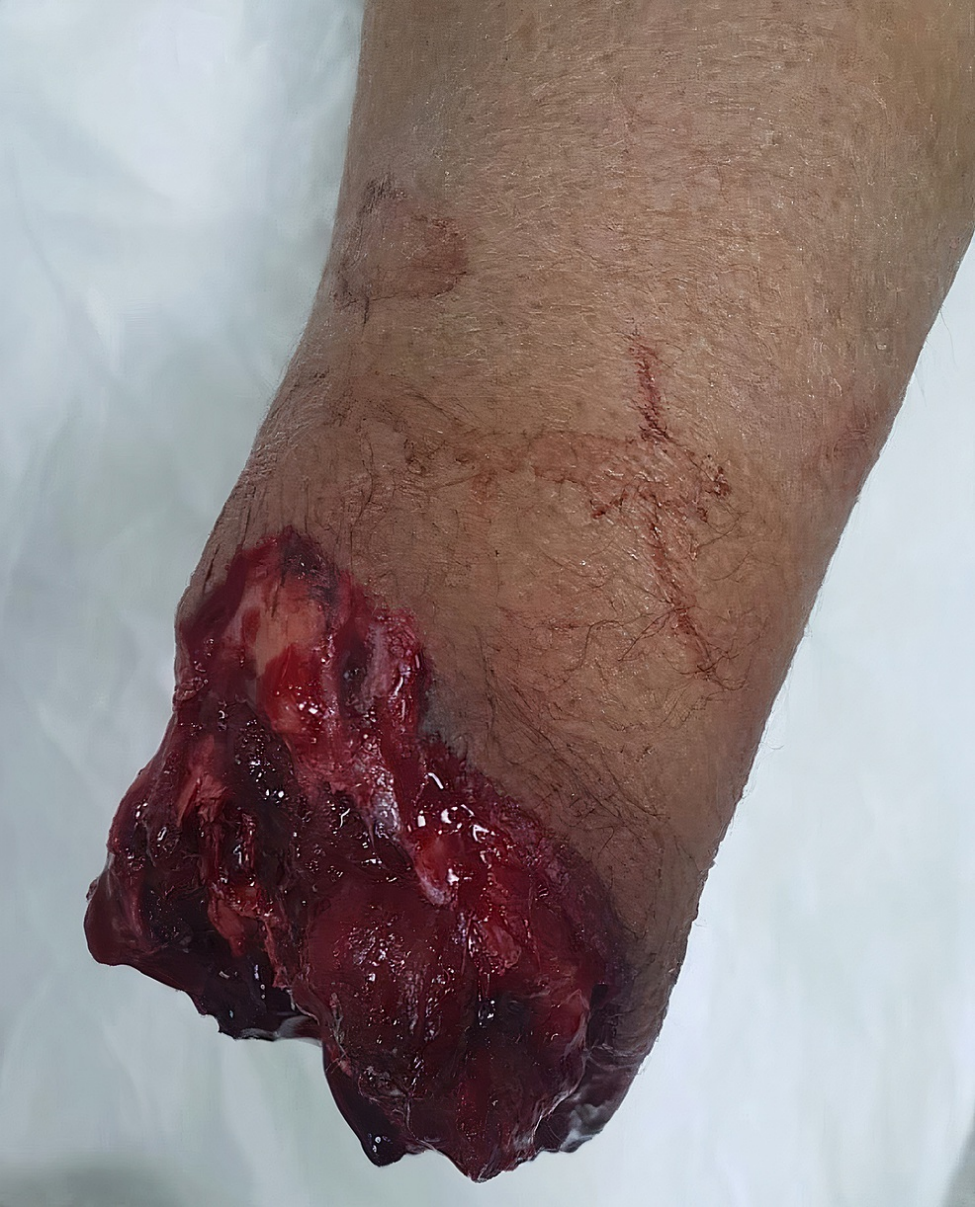
Proximal end of the amputated limb

A 47-year-old right-handed male patient sustained an injury when his left hand was cut off while using an electric saw machine. The patient’s coworkers tried to stop the bleeding with napkins and a cotton scarf covering the injury site. The amputated part was placed in a plastic bag, and the patient was taken to a local primary health clinic at Al-Qaseem province. The amputated part was wrapped and placed in ice inside a container. His colleagues transferred the patient to the emergency department at King Saud Medical City in Riyadh six hours after the accident. Upon arrival, the patient was conscious and oriented. Vital signs were stable. His left forearm stump was covered with blood-soaked fabric (Figure 1). The patient shortly became unstable and was resuscitated with IV fluids. X-rays were obtained, revealing an amputation of both the radius and ulna at the distal third part with a mangled extremity severity score of 6, which in return warrants replantation (Figure 2).

The amputated part was gently and thoroughly irrigated with normal saline at a temperature of 38°C and carefully examined. A decision to salvage the hand was taken jointly with the orthopedic team, and consent was obtained from the patient after explaining the advantages and disadvantages of the operation. He was then immediately taken for surgery under general anesthesia (GA). The raw area on the amputated hand was covered with saline-soaked gauze and placed in sterile polythene. After rigorous washing with normal saline, crushed and devitalized tissue were debrided from both the amputated part and distal end of the forearm. A shunt between the proximal and distal vessels was used to reduce ischemia time and wash out all accumulated toxins. Afterward, the arteries, veins, nerves, and tendons were tagged. With the help of the orthopedic team, bones on the amputated part were shortened by almost 1 cm; unfortunately, X-rays were not obtained because direct reduction was done. Four K-wires were passed through both radius and ulna for fixation. Both radial and ulnar arteries were freshened, and bleeding was observed. Repair of the radial artery was performed, and backflow was established around six hours after injury. Subsequently, the ulnar artery and cephalic vein on the dorsum aspect were repaired. All vascular anastomoses and nerve repair were done with 10-0 Nylon; end-to-end repair of the nerves was amenable because of the sharp nature of the injury under the operating microscope (Zeiss Opmi Pentero, Zeiss, Oberkochen, Germany).

**Figure 3:**
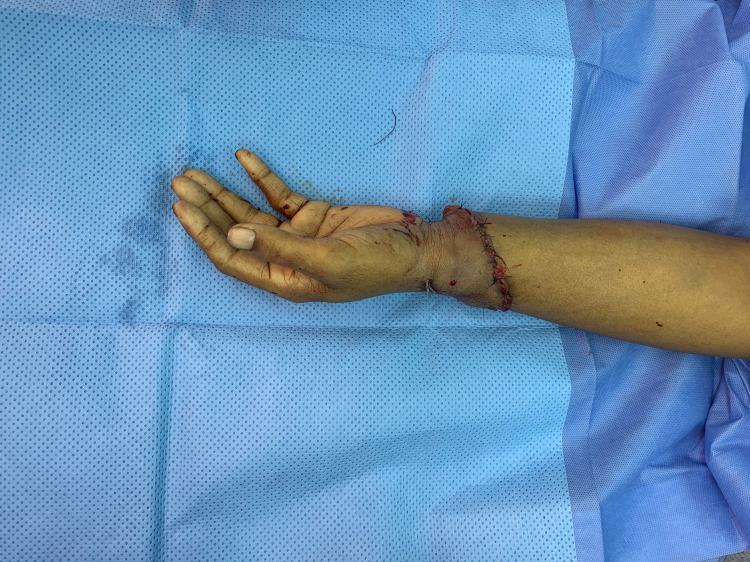
Post replantation

Reaching the end of the procedure, the tourniquet was elevated to check for capillary refill and distal pulses; then, the repair of the flexor tendons was completed. As for the extensor tendons, a mass repair fashion was used to prevent further unwarranted delay of surgery. Tendon repair was done using 5-0 Nylon using surgical loops. Wounds were sutured in layers and were covered with dressings, and a back slab was applied. The surgery lasted 12 hours, and the patient recovered from anesthesia uneventfully (Figure 3). The patient received two units of PRBCs due to low hemoglobin readings and a single dose of low-molecular-weight heparin intraoperatively. Postoperatively, the patient received aspirin and antibiotics for two weeks and was closely monitored in the surgical ICU for any complications. Team members did daily dressings and regular checkups during on-calls. Distal pulse oximeter readings were ranging between 95% and 97% O2 saturation with audible and palpable distal pulses and capillary refill of less than two seconds.

**Figure 4:**
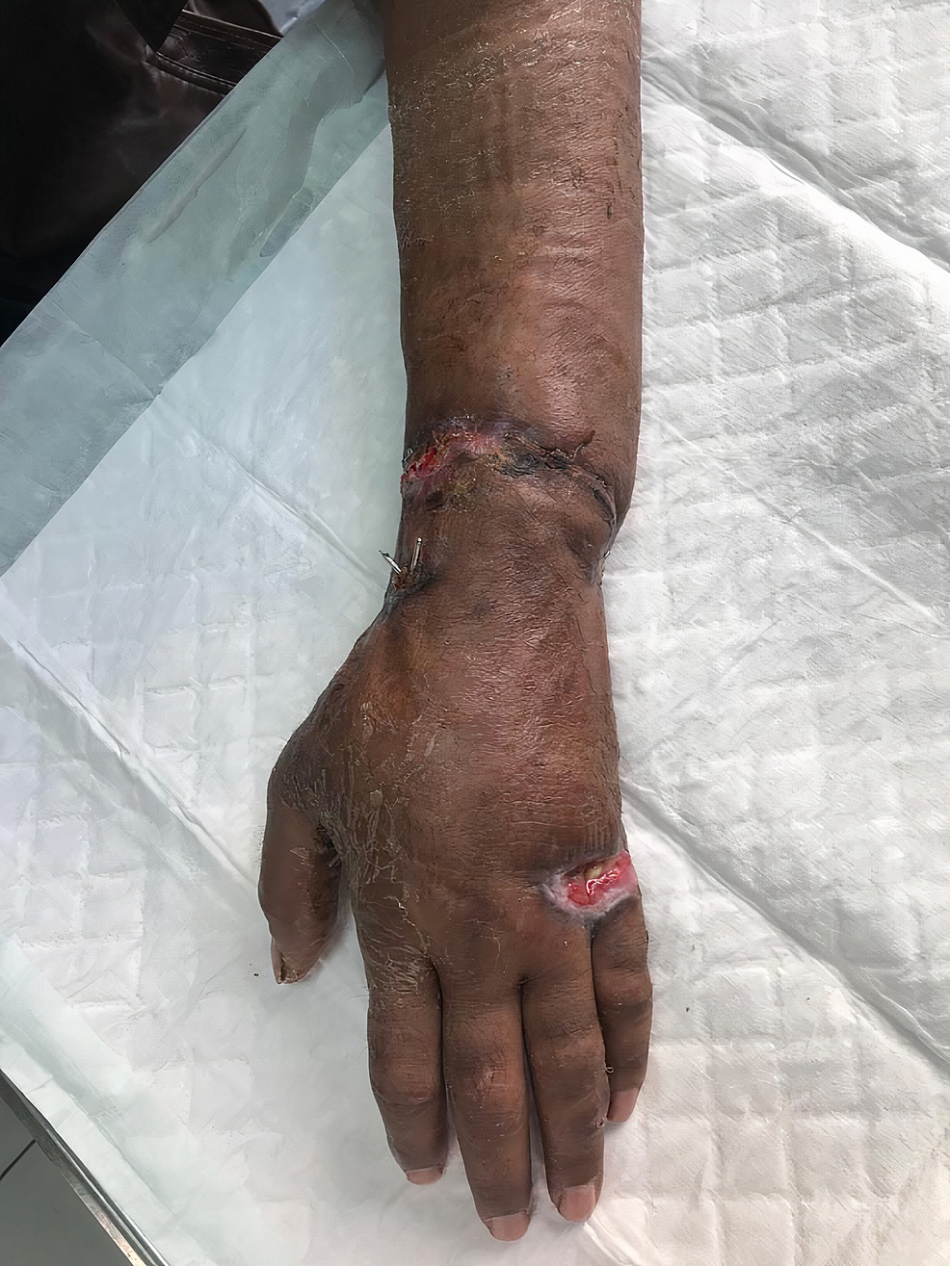
Three months’ visit to the clinic

After two weeks, the patient was shifted to the general ward for further assurance and then discharged safely. The patient had regular follow-ups in our center to assess wounds and function. The first appointments, which were every two weeks, showed minimal advancement in function. However, there was a significant reduction in swelling, and the wounds looked healthy with no local signs of infection. Over the next six months, the patient showed advancement in function in the picture of distal phalanx flickering movements (Figure 4). The patient continued physiotherapy at his hometown, and longer follow-up appointments were given to monitor improvement.

## Discussion

Replantation involves the reconstitution of all the separated components of an extremity, including anastomosis of the arterial inflow and venous return. Amputations can be classified by site of injury, type of injury, degree of contamination, and associated local injuries. In upper limb amputations, the major determining factor in classifying them into major and minor is muscle bulk. Amputations located at the level of the arm and forearm are considered significant in comparison to finger amputations; this is due to the low tolerance of the muscles to ischemia. The ultimate result of replantation is to restore form and function. The function is superior to form as it significantly impacts the individual’s life and career. It is crucial to know the steps and recommendations to reserve the amputated part and deliver it to the hospital in the best condition possible. Optimum care of the amputated part can be achieved by placing it in a plastic bag kept in ice, which helps reduce warm ischemia time. Pressure and compression is the ideal way to manage bleeding vessels. It is advised to trim down any excess tissue remaining at the injury site. The severed part is to be wrapped and kept as clean as possible until delivered safely to the hospital, with limb elevation at all times. From the arrival of the patient at the emergency department, prompt admission and proper management is mandatory. A multidisciplinary team should be involved to facilitate a successful replantation [9]. The appropriate measures taken to establish an optimal outcome involve preoperative, intraoperative, and postoperative care [10]. Preoperative care includes administration of fluids, administration of antibiotics, and warming of the patient to prevent hypothermia and vasoconstriction. Intraoperative steps include tagging and protecting the nerves, vessels, and tendons; bone shortening to reduce the tension across the neurovascular bundle; K-wire fixation; restoring good arterial blood flow; ultimate repair of at least one artery and two veins; and repair of all tendons if possible. Postoperative care includes administering adequate fluids; warming the patient’s room to prevent hypothermia and vasospasm; using anticoagulants; close monitoring for signs of reperfusion, compartment syndrome, or arterial and venous insufficiency; and detecting and treating any surgical sight infection or postoperative complications. To ensure a successful replantation, every step counts from the start of the amputation to shifting the patient back to the ward. Physiotherapy and occupational therapy play a significant role in helping the patient regain as much function as possible [11].

## Conclusions

To conclude, we report a rare case of hand amputation successfully replanted with the repair of two arteries and one vein. However, we advise the restoration of one artery and two veins as it increases the chance of a successful replantation. Our patient achieved improvement in both shape and function. The regular follow-ups achieved this improvement at our clinic and physiotherapy sessions. At first, minimal function was observed. However, within six months, the patient showed good advancement in function. The patient continues the same management to reach the maximum possible functional outcome. We want to propagate the importance of preoperative care, specifically proper care of the amputated part, and minimize the patient’s commute time to the hospital. Replantation is major surgery with no guaranteed results; therefore, ensuring as many optimal conditions are met, from the time of the incident to the postoperative phase, is essential in restoring the top form and function.
